# Supplementary material for: γδ T cell receptor recognition of CD1d in a lipid-independent manner
Source: Nat Commun. 2025 Dec 29;17:926. doi: 10.1038/s41467-025-67653-0 (PMC12830645; doi:10.1038/s41467-025-67653-0)
Supplement: Supplementary file 1 — Supplementary information [file 41467_2025_67653_MOESM1_ESM.pdf]

Supplementary information

γδ T cell receptor recognition of CD1d in a lipid-independent manner

Michael T. Rice[1], Sachith D. Gunasinghe[1,¶], Chhon Ling Sok[1], Mengqi Pan[1], Chan-Sien Lay[1], Benjamin S. Gully[#1,¶] and Jamie Rossjohn[#1,2]

Supplemental Data

Supplemental Figures 1-11

Supplemental Tables 1-4

Supplementary Table 1 : γδ TCRs used within this study. γδ TCRs: 2, 3, 6, 7 & 8 were previously identified from PBMC derived CD3+ γδ T cells from ref 19.

TCR	Donor	Chain Usage	CDR3γ	CDR3δ
2	3	Vδ1-Vγ5	CATWDRLYYKKLF	CALGVWGDKLIF
3	3	Vδ1-Vγ2	CATWDGLSYKKLF	CALGELLVRSSLTAQLFF
6	5	Vδ1-Vγ9	CALWEARPFYYKKLF	CALGELWGFPNRDKLIF
7	6	Vδ1-Vγ5	CATWDRPEANYYKKLF	CALGEPFLRSLIWEYTDKLIF
8	6	Vδ1-Vγ5	CATWDALAKLF	CALGETFLPSLGGWTDKLIF
9C2		Vδ1-Vγ5	CATWDRGNPKTHYYKKLF	CALGDPGGLNTDKLIF
DP10.7		Vδ1-Vγ4	CATWDEKYYKKLF	CALGEPSYWGFPRTTRVIF

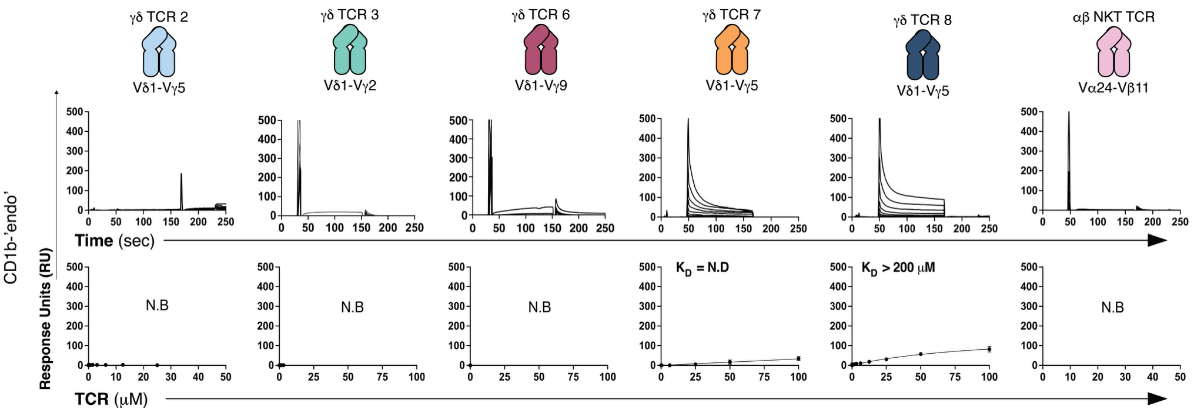

Supplementary Figure 1: γδ TCR Affinity Measurements. Affinity measurement analysis of γδ TCRs 2, 3 & 6, 7, 8 and the αβ NKT TCR determined by SPR against CD1b-'endo'. Sensograms are representative of a single dilution series. Error bars denote S.E.M. N=2 independent experiments, performed in duplicate. N.D = not determined.

Supplementary Table 2. SEC-SAXS Data analysis.

Sample	Concentration (mg/mL)	Radius of Gyration, R_g (Å)	Maximum Dimension, D_{max} (Å)	Molecular Mass, from V_p (kDa)	Oligomeric state	Observed Stoichiometry (TCR:CD1d)	Calculated Molecular Mass (kDa)
γδ TCR 2	8.55	43.4 ± 0.36	170	95.9	1.8	2:0	52.08
γδ TCR 3	8	29.84 ± 0.19	114	43.6	0.83	1:0	52.37
γδ TCR 6	7.85	30.3 ± 0.20	118	46.2	0.8	1:0	54.27
γδ TCR 8	7.25	40.05 ± 0.24	149	85.3	1.5	2:0	52.79
CD1-endo	7	27.14 ± 0.02	118	51.8	0.84	0:1	43.73
γδ TCR 2 CD1d-'endo'	8	43.58 ± 0.36	163	104	1.09	1:1	95.81
γδ TCR 3 CD1d-'endo'	5	40.81 ± 0.31	164	76.5	0.79	1:1	96.09
γδ TCR 6 CD1d-'endo'	7.85	40.47 ± 0.18	155	80	0.83	1:1	96.52
γδ TCR 7 CD1d-α-GalCer	4	37.07 ± 0.52	157	67.1	0.69	Un-complexed	97.30
γδ TCR 8 CD1d-'endo'	8	40.86 ± 0.19	157	88.2	0.88	Un-complexed	100.60

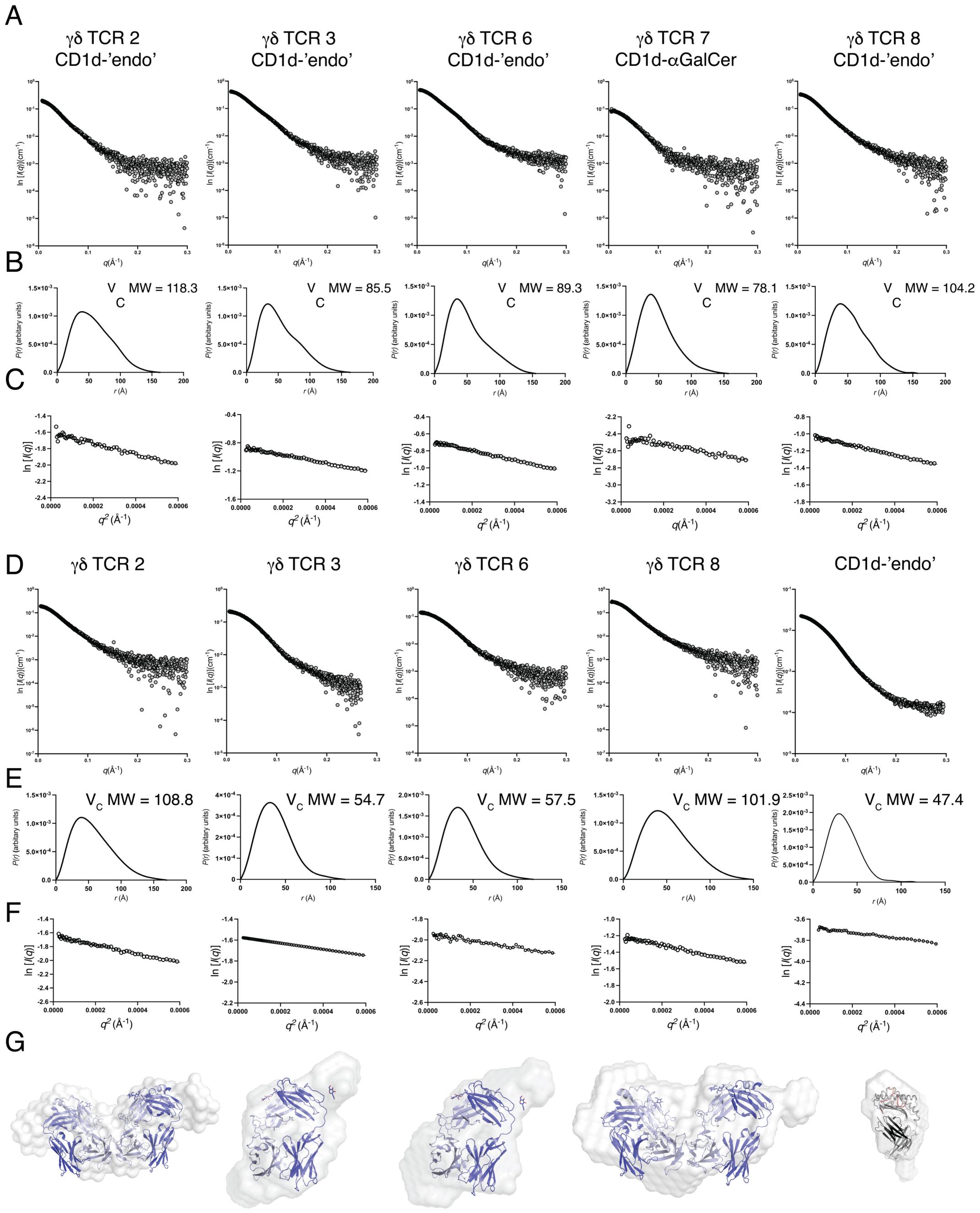

Supplementary Figure 2: γδ TCR SEC-SAXS data collection. SEC-SAXS scattering profile (**A**), P(r) distribution plot (**B**), Guinier analysis (**C**) and *ab initio* model (**D**) for γδ TCR 2, γδ TCR 3, γδ TCR 6 and γδ TCR 8 complexed with CD1d-'endo' or CD1d-α-GalCer. (**D-G**) SEC-SAXS monomer samples, scattering profile (**D**), P(r) plot (**E**), Guinier analysis (**F**) and *ab initio* reconstruction (**G**). Data was processed in BioXTSAS RAW software suite and GraphPad Prism 10.3.1. The 9C2 γδ was aligned to the *ab initio* model, colored white, via DAMMIF, with γ/δ chains in light and dark blue respectively. CD1d shown in dark grey and β_2M in black. V_C denotes volume of correlation, MW denotes molecular weight.

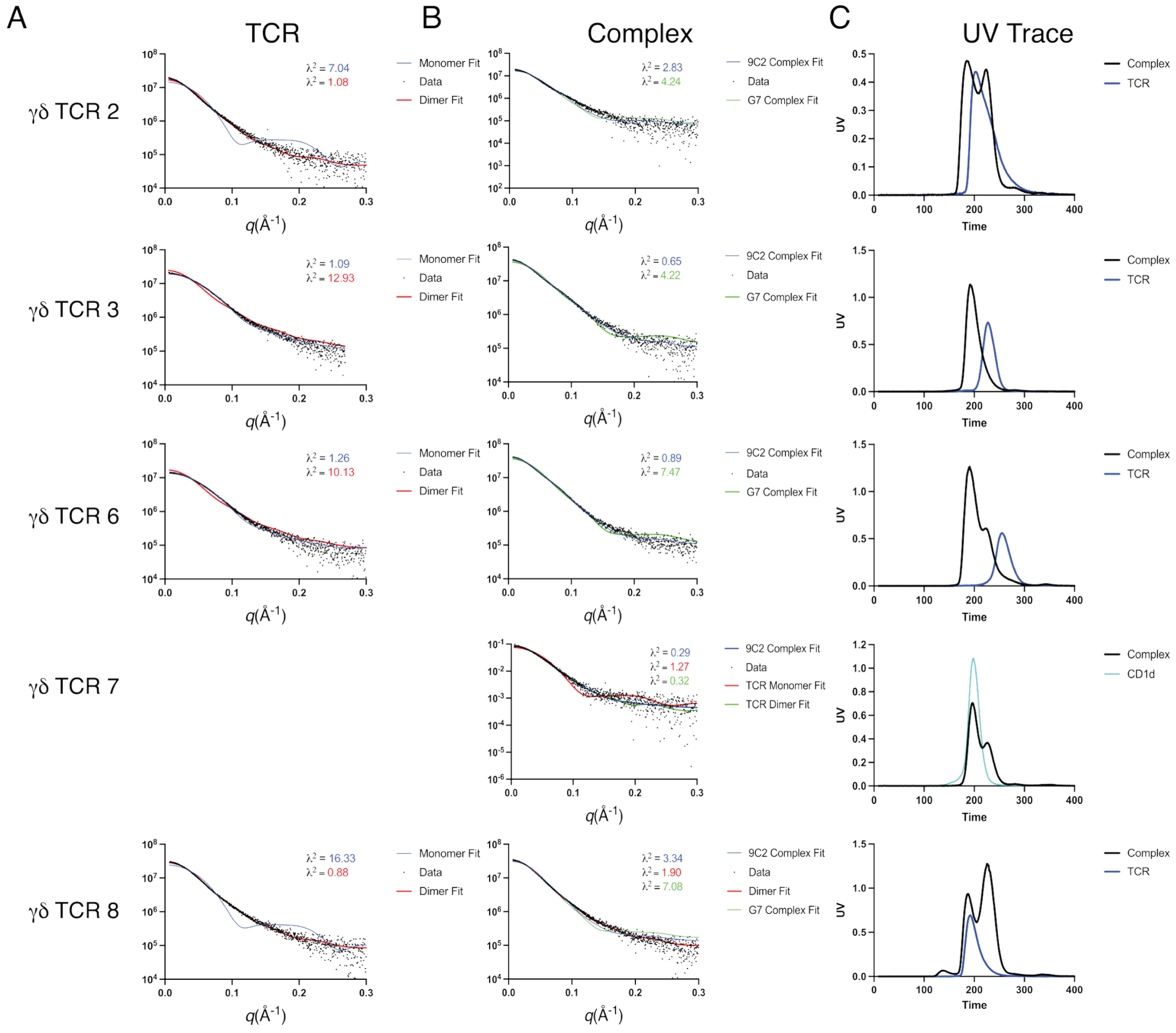

Supplementary Figure 3: SEC-SAXS CRYSOL analysis. CRYSOL analysis of γδ TCR 2, γδ TCR 3, γδ TCR 6 and γδ TCR 8, for TCR alone (**A**) or complexed to CD1d-'endo' (**B**). SEC UV Trace overlay comparing TCR alone and TCR-CD1d-'endo' was used to assess TCR co-complexation (**C**).

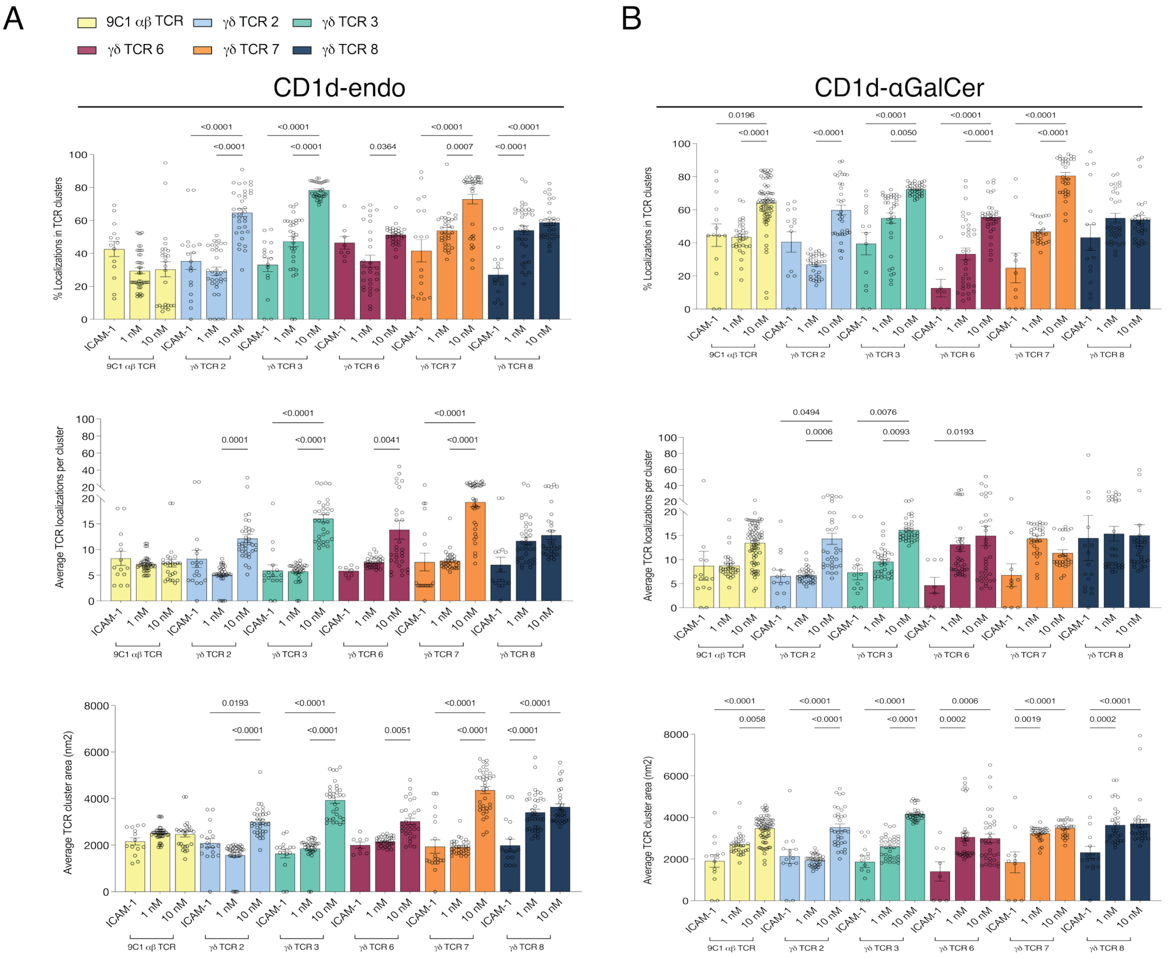

Supplementary Figure 4: Analysis of *d*STORM images from Figure 3. DBSCAN was used to analyse CD1d-endo (**A**) and CD1d-αGalCer (**B**) induced clustering. Data was processed in GraphPad Prism 10.3.1. Statistical analysis was performed using one-way ANOVA with Tukey's multiple comparisons test against ICAM-1 controls. Error bars represent S.E.M.

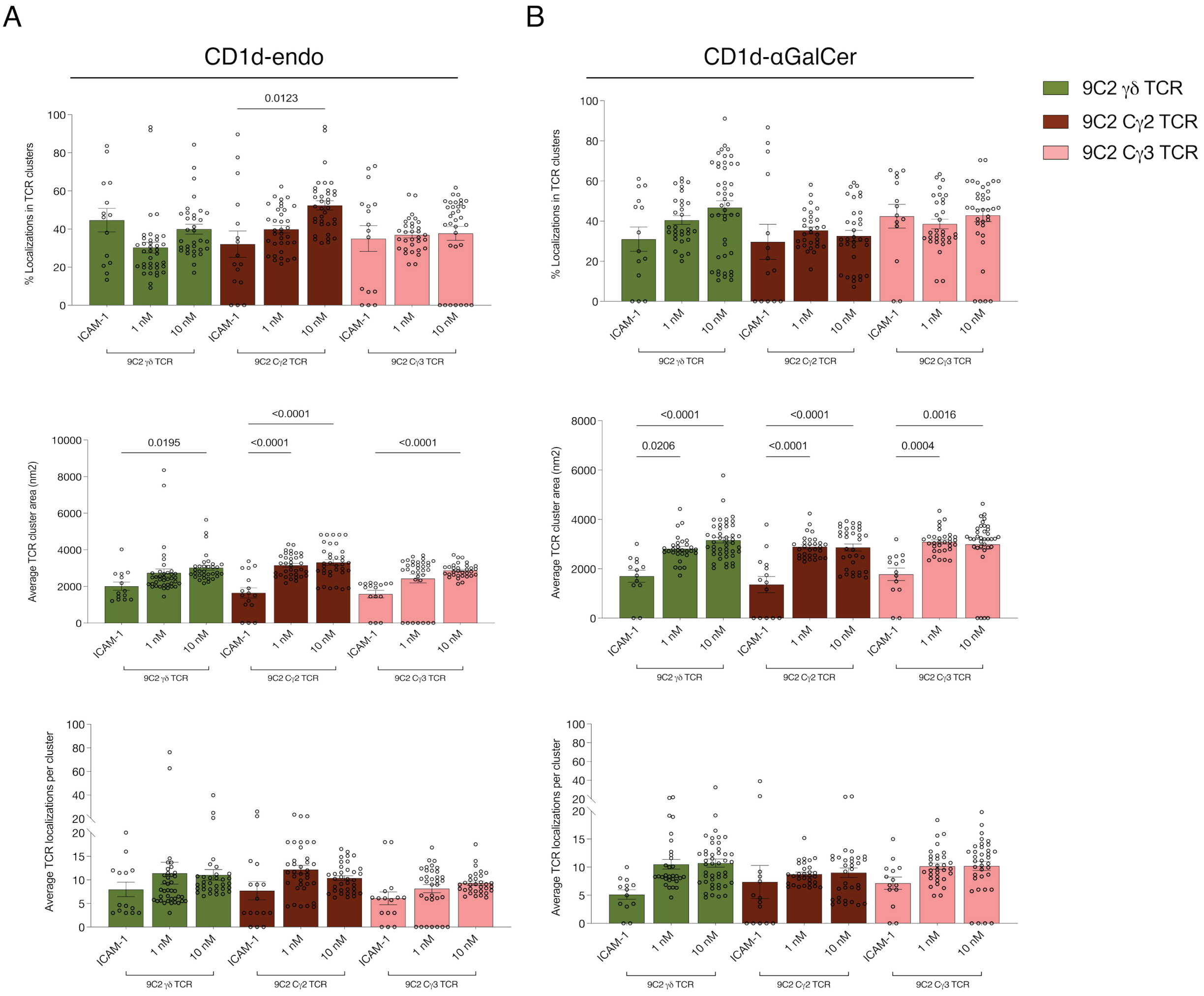

Supplementary Figure 5: Analysis of *d*STORM images from Figure 4. DBSCAN was used to analyse CD1d-endo (**A**) and CD1d-αGalCer (**B**) induced clustering. Data was processed in GraphPad Prism 10.3.1. Statistical analysis was performed using one-way ANOVA with Tukey's multiple comparisons test against ICAM-1 controls. Error bars represent S.E.M.

Supplementary Table 3. Data collection and refinement statistics

	γδ TCR 2-CD1d-‘endo’
**Data collection**	
Temperature	100K
Resolution (Å)	46.65-2.86 (3.02-2.86)
Space group	P 43 21 2
Cell dimensions	
a, *b*, *c* (Å)	178.09, 178.09, 75.43
	$\alpha=\beta=\gamma=90°$
No. of observations	296948 (43221)
No. of unique observations	28516 (4072)
$I / \sigma I$	16.4 (2.3)
Completeness (%)	99.8 (98.7)
Multiplicity	10.4 (10.6)
R_{merge}	0.106 (1.058)
R_{pim}	0.049 (0.491)
CC 1/2	0.999(0.750)
**Refinement**	
R_{work} / R_{free}	20.65/24.97
No. atoms	
Protein	6400
Ligand/ion	142
B-factors	
Protein	84.08
Ligand/ion	108.61
R.m.s. deviations	
Bond lengths (Å)	0.005
Bond angles (°)	0.938
Ramachandran (%)	
Favoured	97.20
Allowed	2.8
Outliers	0

*Values in parentheses are for highest-resolution shell.

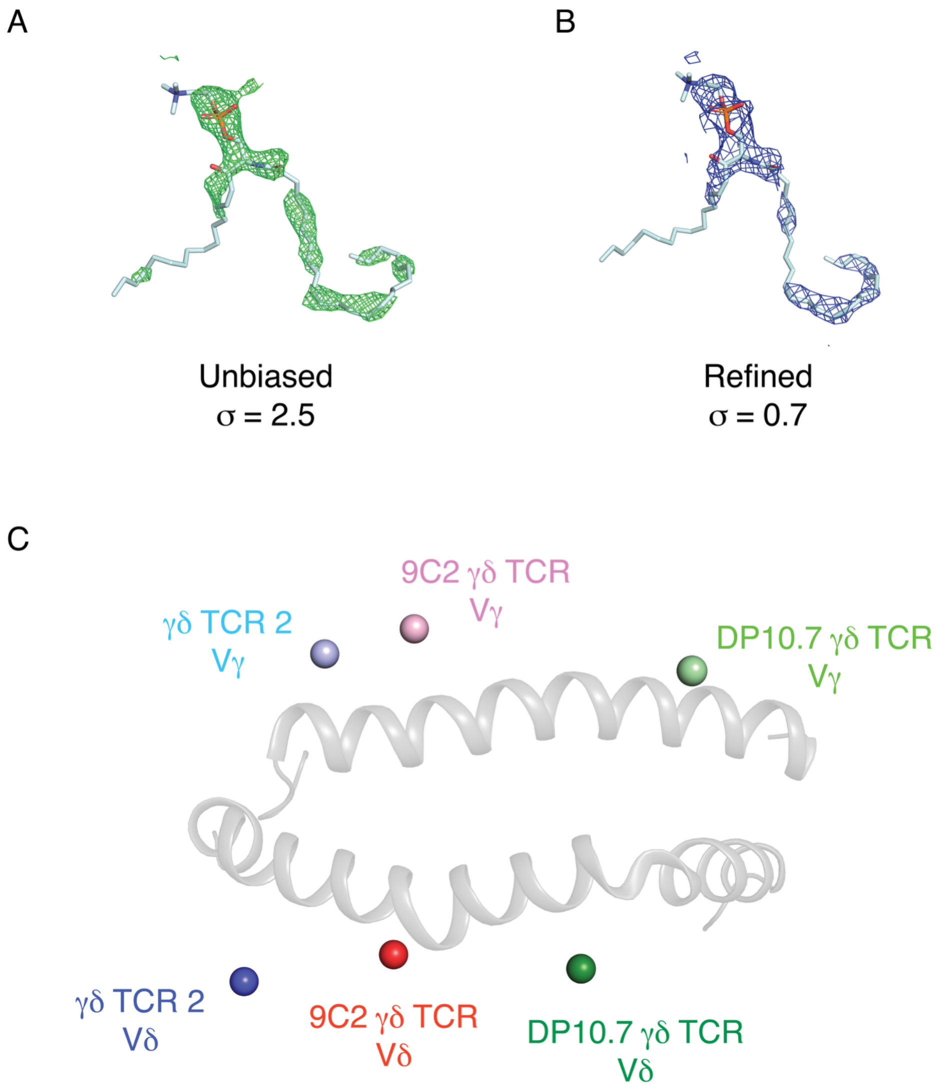

Supplementary Figure 6: Electron density for γδ TCR-CD1d-endo lipid antigen. Unbiased (**A**) and refined (**B**) density for the lipid antigen built into the antigen presenting cleft of CD1d-endo. The sphingomyelin lipid antigen built into the density is shown as sticks. (**C**) Variable domain COM for γδ TCR 2, 9C2 γδ TCR and DP10.7 γδ TCR. Vγ/Vδ shown in light and dark variants of blue, red and green. CD1d shown in grey.

Supplementary Table 4. γδ TCR 2 contacts with CD1d-'endo'.

TCR Gene	TCR Residues	CD1d Residues	Bond Type
CDR1γ	Ile31	Trp153	VDW
	Phe34	His68, Ile69	VDW
	Tyr35	Gln61, Glu64, Thr65, His68	VDW
FWγ	His37	Gln61	
	$Arg49^{NH1}$	$Gln61^{O\varepsilon1}$	HB
	Arg49	Gln61	VDW
	Tyr52	Gln61	VDW
CDR2γ	$Asp54^{O\delta1,2}$	$His68^{N\varepsilon2}$	HB
	Ser56	His68	VDW
	Asn57	Arg71	VDW
CDR3γ	Arg103	Trp153, Glu156	VDW
	Leu104	Glu156,Trp160	VDW
	Tyr105	Thr65, Trp160	VDW
CDR1δ	Trp33	Glu175	VDW
	Ser34	Gln168	VDW
CDR3δ	Val98	Gln62	VDW
	Trp99	Phe58, Gln62, Trp160, Thr165, Gln168	VDW
	$Trp99^{N\varepsilon1}$	$Gln168^{O\varepsilon1}$	HB
	$Trp99^{O}$	$Gln62^{N\varepsilon2}$	HB
	$Gly100^{O}$	$Gln61^{N\varepsilon2}$	HB
	Gly100	Gln61	VDW
	Asp101	Ser59, Gln61	VDW

HB: Hydrogen bond, VDW: Van der Waals, SB: salt bridge. Cut-off at 4 Å for VDW interactions and SB, 3.5 Å for HB.

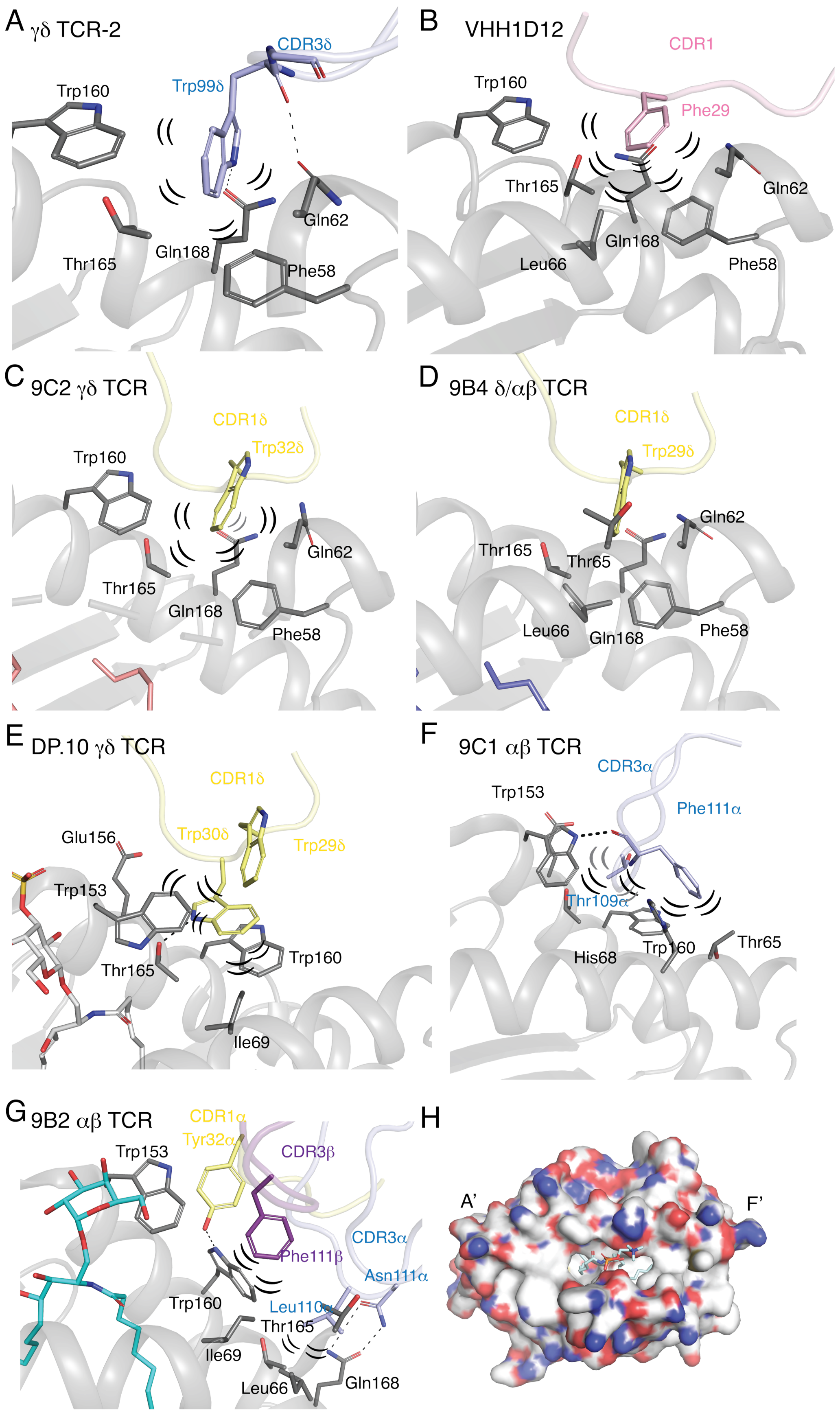
A γδ TCR-2
CDR3δ
Trp99δ
Trp160
Gln62
Gln168
Thr165
Phe58
B VHH1D12
CDR1
Phe29
Trp160
Thr165
Gln62
Gln168
Leu66
Phe58
C 9C2 γδ TCR
CDR1δ
Trp32δ
Trp160
Gln62
Thr165
Gln168
Phe58
D 9B4 δ/αβ TCR
CDR1δ
Trp29δ
Gln62
Thr165
Thr65
Leu66
Gln168
Phe58
E DP.10 γδ TCR
CDR1δ
Trp30δ
Trp29δ
Glu156
Trp153
Thr165
Trp160
Ile69
F 9C1 αβ TCR
CDR3α
Trp153
Phe111α
Thr109α
His68
Trp160
Thr65
G 9B2 αβ TCR
CDR1α
Tyr32α
CDR3β
Trp153
CDR3α
Phe111β
Asn111α
Trp160
Leu110α
Thr165
Ile69
Leu66
Gln168
H
A'
F'

Supplementary Figure 7: A conserved CD1d binding motif. Comparison of interactions between the A' of CD1d and γδ TCR 2 (**A**), VHH1D12 (**B**), 9C2 (**C**), DP.10 (**D**), 9B4 (**E**), 9C1 (**F**) and 9B2 (**G**) . Surface charge of CD1d (**F**). CD1d is shown in dark grey, CDR1 α/δ in yellow, CDR3 α/δ in light blue and CDR3γ in purple.

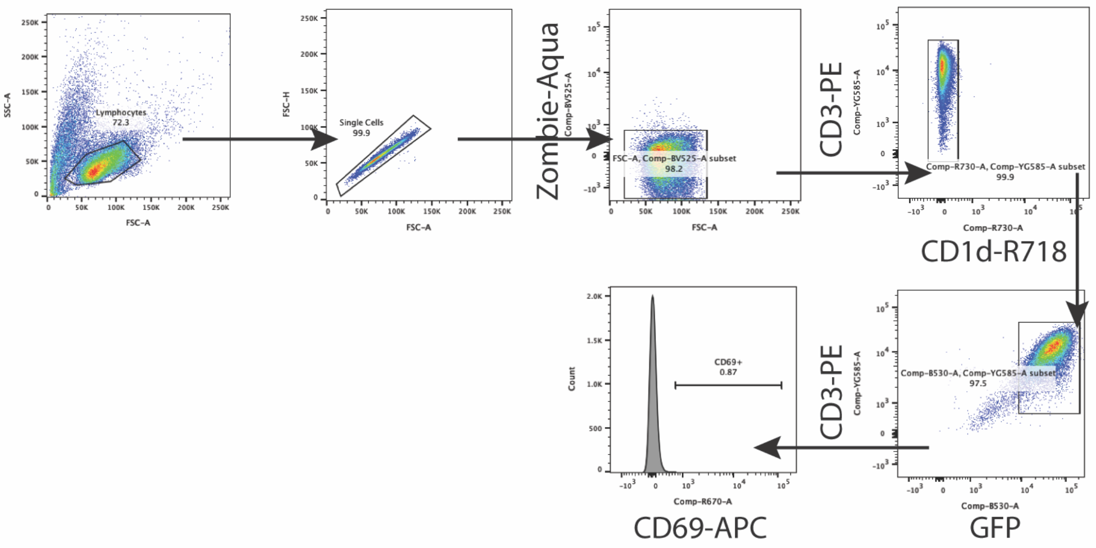

Supplementary Figure 8: T cell activation gating strategy. Gating strategy utilised to identify Jurkat.76 TCR activation.

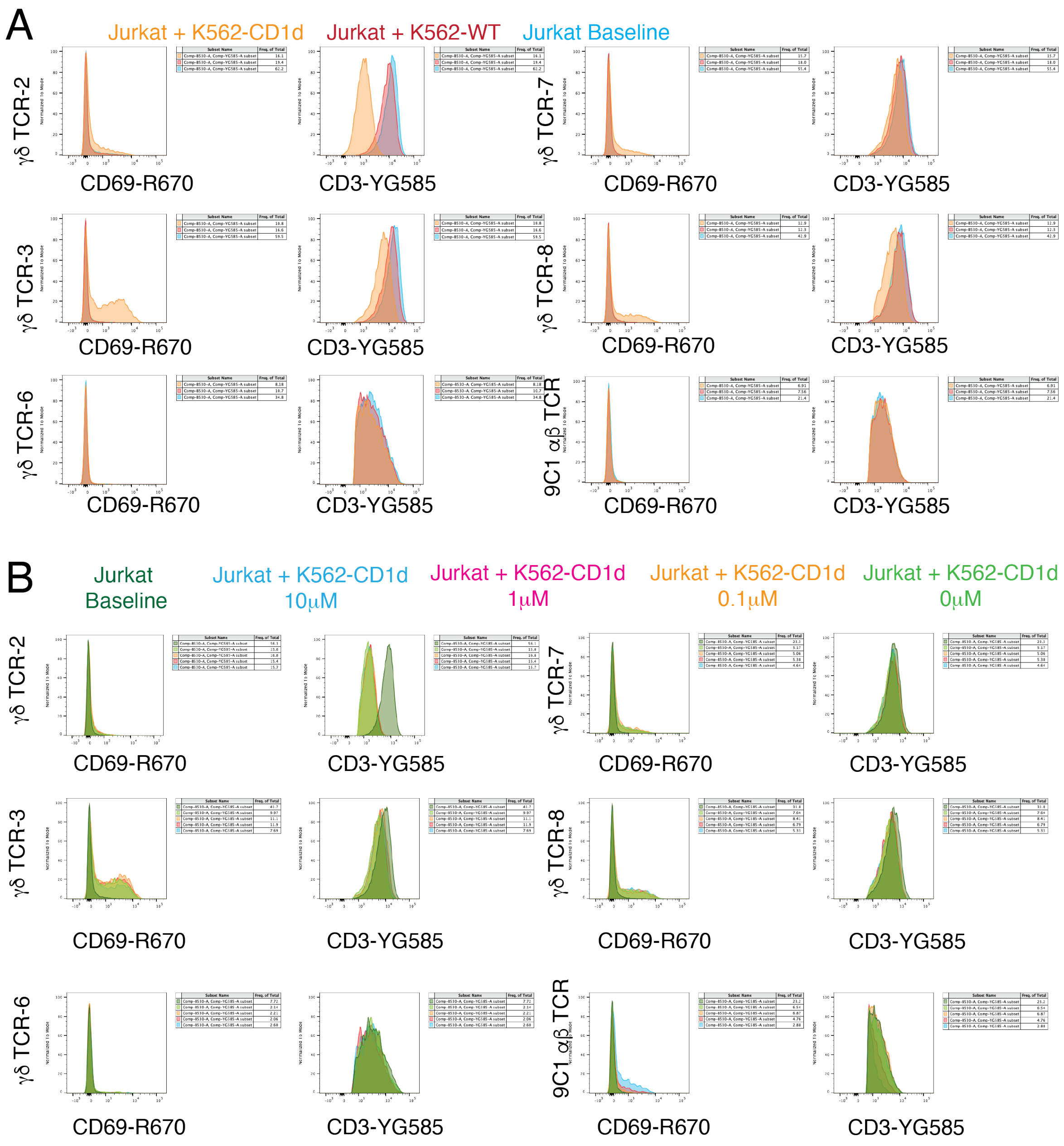

Supplementary Figure 9: Representative Histograms from CD1d induced activation. (A) CD69 upregulation and CD3 downregulation from Figure 2A following γδ TCR Jurkat cells activation against K562-WT or K562-CD1d cells following 16 hours. (B) CD69 upregulation and CD3 downregulation after 16 hours from Figure 2B following γδ TCR Jurkat activation against serial dilution of α-GalCer (μM) with K562-CD1d cells. Analysis was performed utilising the gating strategy seen in Supplementary Figure 8; lymphocytes, single cells, live cells, CD1d$^-$CD3$^+$, CD3$^+$GFP$^+$. Histograms are representative of a single condition, data was generated from two independent experiments performed in duplicate.

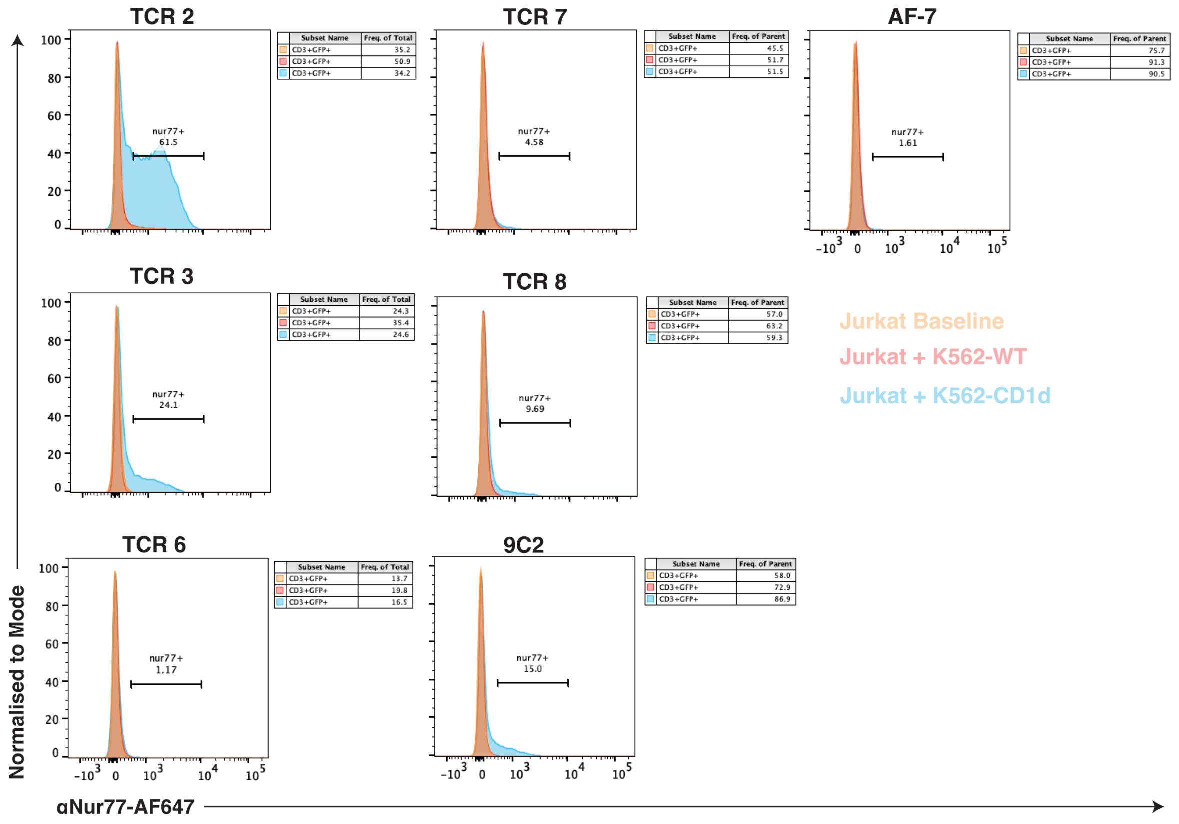

Supplementary Figure 10: Representative histograms of Nur77 upregulation from Figure 2C. Nur77 upregulation after 2 hours from Figure 2B following γδ TCR Jurkat activation against K562-WT or K562-CD1d cells. Analysis was performed utilising the gating strategy seen in Supplementary Figure 8; lymphocytes, single cells, live cells, $CD1d^{-}CD3^{+}$, $CD3^{+}GFP^{+}$. Gates for Nur77 are depicted on the histogram within the bracket. Histograms are representative of a single condition, data was generated from at least two independent experiments performed in duplicate.

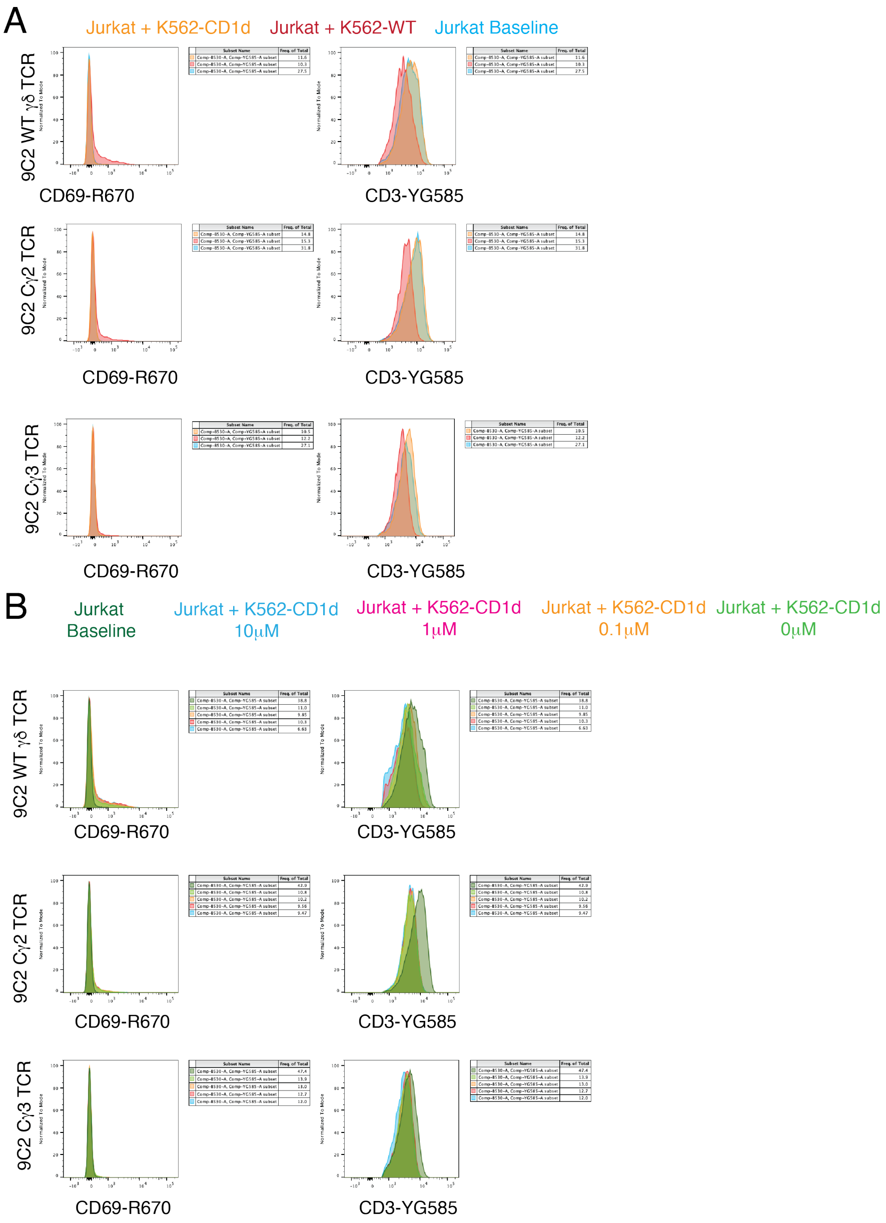

Supplementary Figure 11: Representative histograms of CD1d activation on Cγ alleles. **(A)** CD69 upregulation and CD3 downregulation for Figure 4 A 'endo' activation following 16 hours for 9C2 Cγ allele transduced Jurkat cells. Activation was measured against K562-WT and K562-CD1d cells. **(B)** CD69 upregulation and CD3 downregulation for Figure 4 B for 9C2 Cγ allele transduced Jurkat cells following serial dilution of α-GalCer (μM) with K562-CD1d cells after 16 hours. Analysis was performed utilising the gating strategy seen in Supplementary Figure 8; lymphocytes, single cells, live cells, CD1d$^-$CD3$^+$, CD3$^+$GFP$^+$. Histograms are representative of a single condition, data was generated from two independent experiments performed in duplicate wells.
