# Supplementary material for: γδ T cell receptor recognition of CD1d in a lipid-independent manner
Source: Nat Commun. 2025 Dec 29;17:926. doi: 10.1038/s41467-025-67653-0 (PMC12830645; doi:10.1038/s41467-025-67653-0)
Supplement: Supplementary file 3 — Reporting Summary [file 41467_2025_67653_MOESM3_ESM.pdf]

nature portfolio

Corresponding author(s): Rossjohn

Last updated by author(s): 15th Oct 2025

Reporting Summary

Nature Portfolio wishes to improve the reproducibility of the work that we publish. This form provides structure for consistency and transparency in reporting. For further information on Nature Portfolio policies, see our Editorial Policies and the Editorial Policy Checklist.

Statistics

For all statistical analyses, confirm that the following items are present in the figure legend, table legend, main text, or Methods section.

n/a	Confirmed	
☐	☒	The exact sample size (*n*) for each experimental group/condition, given as a discrete number and unit of measurement
☐	☒	A statement on whether measurements were taken from distinct samples or whether the same sample was measured repeatedly
☐	☒	The statistical test(s) used AND whether they are one- or two-sided<br>*Only common tests should be described solely by name; describe more complex techniques in the Methods section.*
☒	☐	A description of all covariates tested
☐	☒	A description of any assumptions or corrections, such as tests of normality and adjustment for multiple comparisons
☐	☒	A full description of the statistical parameters including central tendency (e.g. means) or other basic estimates (e.g. regression coefficient) AND variation (e.g. standard deviation) or associated estimates of uncertainty (e.g. confidence intervals)
☐	☒	For null hypothesis testing, the test statistic (e.g. *F*, *t*, *r*) with confidence intervals, effect sizes, degrees of freedom and *P* value noted<br>*Give P values as exact values whenever suitable.*
☒	☐	For Bayesian analysis, information on the choice of priors and Markov chain Monte Carlo settings
☒	☐	For hierarchical and complex designs, identification of the appropriate level for tests and full reporting of outcomes
☒	☐	Estimates of effect sizes (e.g. Cohen's *d*, Pearson's *r*), indicating how they were calculated

Our web collection on statistics for biologists contains articles on many of the points above.

Software and code

Policy information about availability of computer code

Data collection	For T cell clustering experiments, dSTORM was used.
Data analysis	MATLAB as outlined in Pageon et al 2016. https://github.com/PRNicovich/ClusDoC was used to analyse clustering experiments.

For manuscripts utilizing custom algorithms or software that are central to the research but not yet described in published literature, software must be made available to editors and reviewers. We strongly encourage code deposition in a community repository (e.g. GitHub). See the Nature Portfolio guidelines for submitting code & software for further information.

Data

Policy information about availability of data

All manuscripts must include a data availability statement. This statement should provide the following information, where applicable:

- Accession codes, unique identifiers, or web links for publicly available datasets
- A description of any restrictions on data availability
- For clinical datasets or third party data, please ensure that the statement adheres to our policy

Protein Data Base - 9OX4.

Research involving human participants, their data, or biological material

Policy information about studies with human participants or human data. See also policy information about sex, gender (identity/presentation), and sexual orientation and race, ethnicity and racism.

Reporting on sex and gender	N/A
Reporting on race, ethnicity, or other socially relevant groupings	N/A
Population characteristics	N/A
Recruitment	N/A
Ethics oversight	N/A

Note that full information on the approval of the study protocol must also be provided in the manuscript.

Field-specific reporting

Please select the one below that is the best fit for your research. If you are not sure, read the appropriate sections before making your selection.

[x] Life sciences [ ] Behavioural & social sciences [ ] Ecological, evolutionary & environmental sciences

For a reference copy of the document with all sections, see nature.com/documents/nr-reporting-summary-flat.pdf

Life sciences study design

All studies must disclose on these points even when the disclosure is negative.

Sample size	At minimum all cellular experiments were performed in duplicate with two experimental replicates.
Data exclusions	No data was excluded from analysis
Replication	All experiments performed were reproducible.
Randomization	Randomization was not applicable or performed during this study.
Blinding	This was not applicable to the study, all conditions were known.

Reporting for specific materials, systems and methods

We require information from authors about some types of materials, experimental systems and methods used in many studies. Here, indicate whether each material, system or method listed is relevant to your study. If you are not sure if a list item applies to your research, read the appropriate section before selecting a response.

Materials & experimental systems

n/a	Involved in the study	
[ ]	[x]	Antibodies
[ ]	[x]	Eukaryotic cell lines
[x]	[ ]	Palaeontology and archaeology
[x]	[ ]	Animals and other organisms
[x]	[ ]	Clinical data
[x]	[ ]	Dual use research of concern
[x]	[ ]	Plants

Methods

n/a	Involved in the study	
[x]	[ ]	ChIP-seq
[ ]	[x]	Flow cytometry
[x]	[ ]	MRI-based neuroimaging

Antibodies

Antibodies used	All antibodies used within this study are described within the Methods and listed below:<br>Flow Cytometry:<br><br>anti-CD1d-R710 (BD Biosciences, clone CD1d42), anti-CD3-PE (BD Biosciences, #555333), anti-CD69-APC (BD Biosciences, FN50, 555533), Nur77-AF647 (BD Biosciences, 12.14, #566735),<br><br>Immunostaining:

anti-CD3e AF647 (BioLegend, #300416, Clone UCHT1) , anti-pCD3z AF568 (BD Biosciences,K25-407.69, #558402)

Validation

Antibodies used throughout this study are commercially available and widely used throughout the literature. Prior to experimental use, antibodies were tested to ensure they bound the relevant targets.

Eukaryotic cell lines

Policy information about cell lines and Sex and Gender in Research

Cell line source(s)	Jurkat.76 cells,HEK 293S, High-Five Insect cells and K562 cells have been maintained in house for >10 years and the source is unknown. Expi293F cells were purchased from Gibco (#A14527).
Authentication	HEK 293S, High-Five and Expi293F cells have not been validated, however morphologically and functionally resemble the expected phenotype. The Jurkat.76 and K-562 cells have been validated via collaborators.
Mycoplasma contamination	HEK293S, High-Five Insect cells and K562 cells have not been tested for mycoplasma contamination.
Commonly misidentified lines (See ICLAC register)	N/A

Plants

Seed stocks	*Report on the source of all seed stocks or other plant material used. If applicable, state the seed stock centre and catalogue number. If plant specimens were collected from the field, describe the collection location, date and sampling procedures.*
Novel plant genotypes	*Describe the methods by which all novel plant genotypes were produced. This includes those generated by transgenic approaches, gene editing, chemical/radiation-based mutagenesis and hybridization. For transgenic lines, describe the transformation method, the number of independent lines analyzed and the generation upon which experiments were performed. For gene-edited lines, describe the editor used, the endogenous sequence targeted for editing, the targeting guide RNA sequence (if applicable) and how the editor was applied.*
Authentication	*Describe any authentication procedures for each seed stock used or novel genotype generated. Describe any experiments used to assess the effect of a mutation and, where applicable, how potential secondary effects (e.g. second site T-DNA insertions, mosiacism, off-target gene editing) were examined.*

Flow Cytometry

Plots

Confirm that:

- [x] The axis labels state the marker and fluorochrome used (e.g. CD4-FITC).
- [x] The axis scales are clearly visible. Include numbers along axes only for bottom left plot of group (a 'group' is an analysis of identical markers).
- [ ] All plots are contour plots with outliers or pseudocolor plots.
- [x] A numerical value for number of cells or percentage (with statistics) is provided.

Methodology

Sample preparation	Cellular staining is outlined in the methods and listed here below:<br><br>T cell activation was assessed via the upregulation of CD69 and the downregulation of CD3, and Nur77 expression. For CD69 upregulation detection experiments, transduced Jurkat.76 cells were co-incubated with K562 cells for 16 hours at 37C. The co-culture was then harvested, washed in PBS and stained with Zombie Aqua Live/Dead Stain (BioLegend). Cells were then washed with FACS Buffer and stained with anti-CD1d-R710 (BD Biosciences, 1:200), anti-CD3-PE (BD Biosciences, 1:200) and anti-CD69-APC (BD Biosciences, 1:200) antibodies. For Nur77 experiments, transduced Jurkat.76 cells were incubated with K562 cells for 2 hours 37C and then harvested. Cells were then stained with Live/Dead stain and stained with anti-CD1d-R710 (BD Biosciences, 1:200) and anti-CD3-PE (BD Biosciences, 1:200).
Instrument	BD LSR Fortessa
Software	FlowJo, GraphPad Prism
Cell population abundance	Double positive TCR transduced Jurkats were generated as outlined in the Methods. Briefly, CD3+ and GFP+ cells were selected and underwent several rounds of cellular sorting to generate high levels of CD3/GFP expression.
Gating strategy	The gating strategy is demonstrated in the gating strategy Figure. Briefly, FSC/SSC gate was used to select the lymphocyte population, followed by a FSC-H/A gate to select for single cells. Live-single cells were then selected with a FSC-A vs BV525-Zombie Aqua gate. This was followed by a R718 vs GFP gate to remove CD1d+ K562 cells from activation experimental

analysis. CD3-PE+ and GFP+ Jurkat T cells were then selected and analysed for their CD69-APC upregulation.

☒ Tick this box to confirm that a figure exemplifying the gating strategy is provided in the Supplementary Information.
